# A new method for assessing the risk of infectious disease outbreak

**DOI:** 10.1038/srep40084

**Published:** 2017-01-09

**Authors:** Yilan Liao, Bing Xu, Jinfeng Wang, Xiaochi Liu

**Affiliations:** 1 The State Key Laboratory of Resources and Environmental Information System, Institute of Geographic Sciences and Natural Resources Research, Chinese Academy of Sciences, Beijing 100101, China; 2 The Key Laboratory of Surveillance and Early-Warning on Infectious Disease, Chinese Center for Disease Control and Prevention, Beijing 102206, China; 3 Sino-Danish College, University of Chinese Academy of Sciences, Beijing, 100190, China; 4 Sino-Danish Center for Education and Research, Beijing, 100190, China; 5 School of Information Engineering, China University of Geosciences, Beijing 100083, China

## Abstract

Over the past few years, emergent threats posed by infectious diseases and bioterrorism have become public health concerns that have increased the need for prompt disease outbreak warnings. In most of the existing disease surveillance systems, disease outbreak risk is assessed by the detection of disease outbreaks. However, this is a retrospective approach that impacts the timeliness of the warning. Some disease surveillance systems can predict the probabilities of infectious disease outbreaks in advance by determining the relationship between a disease outbreak and the risk factors. However, this process depends on the availability of risk factor data. In this article, we propose a Bayesian belief network (BBN) method to assess disease outbreak risks at different spatial scales based on cases or virus detection rates. Our experimental results show that this method is more accurate than traditional methods and can make uncertainty estimates, even when some data are unavailable.

With the appearance of a variety of new infectious diseases (e.g., H7N9, H5N1, and Ebola) and the renewed prevalence of existing diseases (e.g., dengue fever; hand, foot, and mouth disease; measles; and bacillary dysentery)^1^, the outbreak and prevalence of infectious diseases pose a serious threat to human health and life. Some infectious diseases have high mortality and fatality rates, and some of these diseases lack treatments or vaccines. Therefore, it is important to monitor disease occurrence and to give timely early warnings of potential disease outbreaks. These measures generate sufficient information on disease epidemics, reduce infectious disease outbreaks, and control the effects of the disease on specific populations^2^,^3^. To this end, the detection of abnormal disease distribution sand the accurate assessment of the risks of disease outbreaks are paramount.

Thus far, organizations at the national, state, and local level, including the American Centers for Disease Control and Prevention (CDC)^4^, Germany’s SurvNet@RKI^5^, and the University of Pittsburgh’s (USA) Real-time Outbreak and Disease Surveillance (RODS) Laboratory^6^, have developed various disease surveillance systems to improve public health. These surveillance systems generate a massive amount of data detailing the early detection of disease outbreaks. Generally, the early detection of disease outbreaks has two main goals: identifying the duration, locations, and shapes and sizes of potential disease clusters; and determining whether each of these potential clusters is due to a genuine outbreak or to chance fluctuations in case counts (see the *Handbook of Bio-surveillance*: “spatial and spatial-temporal”). To this end, many new techniques have been introduced over recent decades, including classical time-series models, as well as methods for detecting spatial and spatio-temporal clusters^7^,^8^. For example, since 2004, China’s CDC has established the China Infectious Disease Automated-alert and Response System(CIDARS)^9^. This system uses simple time-aggregation methods, such as control charts^10^, historical limits^11^, and SaTScan^12^ to estimate the risk of infectious disease outbreaks. However, these methods are all retrospective analyses that do not entail prospective studies. Multiple regression, What’s Strange About Recent Events (WSARE)^8^, and Hidden Markov models (HMMs)^13^ have been successfully used to predict further disease outbreaks based on multivariate records. An intelligent system proposed by Jiang *et al*.^8^ can even predict the size and duration of the outbreak and estimate how far the outbreak extends into a particular area. However, the accuracy of these methods is excessively dependent on the availability and quality of risk factor data. In practice, risk factor data are often not collected early enough during disease surveillance, especially in developing countries. Therefore, a Bayesian belief network (BBN)may be used to estimate the probability of infectious disease outbreaks in the case of missing risk factors.

BBNs use a combination of graph theory and probability theory based on Thomas Bayes’theorem^14^. They are graphical models of the probabilistic relationships among a set of variables that can express the mutual dependencies between variables in terms of quality and quantity^15^. As such, BBNs have been used in many fields because of their simplicity and soundness, especially in medical and industrial diagnosis^16^. For this paper, we used a BBN to assess the infectious disease outbreak risk on different spatial scales based on cases or virus detection rates. The experimental results showed that, once the relationship network among disease outbreak risks and factors has been determined, BBN accurately predicts the risk of further infectious disease out breaks even when some selected factors are missing. In addition, BBN is flexible for early warning of the infectious disease under different sceneries.

## Methods

### Bayesian Belief Network (BBN) Model

**Figure 1:**
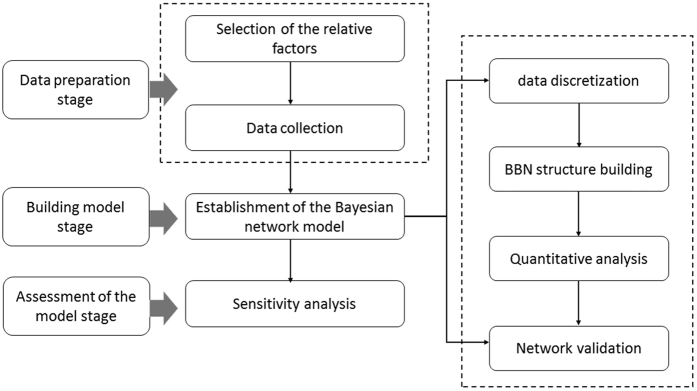
The framework for using Bayesian Belief Network to assess the disease outbreak risk.

A BBN represents the hierarchical and mutual relationships among variables, in contrast to the regression model, which can only represent the outcome variables’ dependencies based on a predictor variable. With the BBN, the joint distribution of all variables is reflected in the directed acyclic graph(DAG), which is a finite directed graph with no directed cycles, where marginal and conditional probabilities can be computed for each node. Therefore, the BBN is more appropriate for prognostic and diagnostic applications. There are many BBN-based risk analysis applications, including analyses of risk in maritime transportation systems’ organizational factors^17^, workplace accidents caused by falls from heights^18^, safety risks for construction projects^19^, and assessing the risks of human neural tube defects^20^. A general framework for infectious disease risk assessment using BBN was designed as shown in Fig. 1. This method comprised three parts: (1) defining the risk level and selecting the risk factors; (2) building the BBN construct; and (3) calculating the joint probability of the target variable in the BBN and assessing the outbreak risk. In this study, the BBN software tool used was BN PowerSoft, which was developed by ChengJie^21^.

### Bayesian belief network construction

A BBN uses a directed acyclic graph (DAG)^15^ to represent the variables and probabilistic causal dependence among the variables. Each variable in the BBN is presented as a node with directed links. The information content of each variable is presented as one or multiple probability distributions. If a variable has no incoming arcs, it is not dependent on any other variables in the model universe, and, if it has parents, it has one probability distribution per combination of possible parent values. Therefore, a reliable relationship network is particularly important to predict disease outbreak risks using BBNs. During the process of Bayesian belief network construction, it is difficult to find a balance between the complexity of the network and the accuracy of probabilistic inference.

Generally, three methods — the expert knowledge method, the network structure learning algorithm, and an integration of these two methods — are applied to build the Bayesian belief network. The expert knowledge method involves building a network by setting nodes, ordering, giving constraints to the structure, and drawing the directed acyclic graph completely according to experts’ comments and the results of lecture review; however, the network structure learning algorithm is based on the independence test proposed by Cheng Jie^21^.

To introduce the approach, we first review the concept of d-separation, which plays an important role in our algorithm. For any three disjoint node sets X, Y, and Z in a belief network, X is said to be d-separated from Y by Z if there is no active undirected path bath between X and Y. A path between X and Y is active if the following conditions are met: (1) every node in the path has head-to-head arrows in Z or has a descendant in Z; and (2) every other node in the path is outside Z. To understand d-separation, we can use an analogy that is similar to the one suggested by (Waidyanatha 2010)^3^. We view a belief network as a network system of information channels. The information flow can pass through an active valve but not an inactive one. When all the valves (nodes) on one undirected path between two nodes are active, we say this path is open. If any valve in the path is inactive, we say the path is closed. When all paths between two nodes are closed given the status of a set of valves (nodes), we say the two nodes are d-separated by the set of nodes. The status of valves can be changed through the instantiation of a set of nodes. The amount of information flow between two nodes can be measured by using mutual information, when no nodes are instantiated, or conditional mutual information, when some other nodes are instantiated.

In information theory, the mutual information of two nodes *X*_*i*_, *X*_*j*_ is defined as


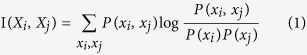


and conditional mutual information is defined as


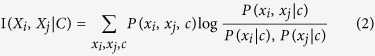


where *X*_*i*_, *X*_*j*_ are two nodes and C is a set of nodes. In our algorithm, we use conditional mutual information as CI tests to measure the average information between two nodes when the statuses of some valves are changed by the condition-set C. When I(*X*_*i*_, *X*_*j*_|C) is smaller than a certain threshold value ε, we say that *X*_*i*_, *X*_*j*_ are d-separated by the condition-set C, and they are conditionally independent.

This algorithm also makes the following assumptions: (1) the database attributes have discrete values and there are no missing values in all the records; (2) the volume of data is large enough for reliable CI tests; and (3) the ordering of the attributes is available before the network construction, i.e., a node’s parents’ nodes should appear earlier in the order.

The algorithm has three phases: drafting, thickening, and thinning. In the first phase, the algorithm computes mutual information of each pair of nodes as a measure of closeness, and creates a draft based on this information. In the second phase, the algorithm adds arcs when the pairs of nodes cannot be d-separated. The result of phase II is an independence map (I-map) of the underlying dependency model. In the third phase, each arc of the I-map is examined using CI tests and will be removed if the two nodes of the arc can be d-separated. The result of Phase III is the minimal I-map.

Phase I: (Drafting)

1) Initialize the graph structure G < V, E> ; where V = nodes (i.e., all attribute fields of the data set), E = {} (E is a set of directed edges joining vertices. No loops of any length are allowed.), and S = Φ, R = Φ.

2) For each pair of nodes (*v*_*i*_, *v*_*j*_) where *v*_*i*_, *v*_*j*_ ∈ *V*, compute mutual information I(*v*_*i*_, *v*_*j*_). For the pairs of nodes that have mutual information greater than a certain small value ε, sort them by their mutual information from large to small, and put them into an ordered set S.

3) Get the first two pairs of nodes in S and remove them from S. Add the corresponding arcs to E. (The direction of the arcs in this algorithm is determined by the previously available nodes ordering).

4) Get the first pair of nodes remaining in S and remove it from S. If there is no open path between the two nodes (these two nodes are d-separated given the empty set), add the corresponding arc to E. Otherwise, add the pair of nodes to the end of an ordered set R.

5) Repeat step 4 until S is empty.

Phase II: (Thickening)

6) Get the first pair of nodes in R and remove it from R.

7) Find a block set that blocks each open path between these two nodes by a set of minimum number of nodes. Conduct a CI test. If these two nodes are still dependent on each other given the block set, connect them by an arc.

8) Go to step 6 until R is empty.

Phase III: (Thinning)

9) For each edge in E, if an open path separate from the current edge remains, it will be brought from E. Then, the independence of the corresponding block set is tested. If the edge and corresponding block set are still correlated, the edge returns to E; otherwise, it is deleted.

Friedman proved theoretically that the method based on independence testing more closely resembles the semantics of the Bayesian networking principle, and it achieves effective results in practice^22^. In addition, for the integration method between the above methods, after constructing the network by the above automatic learning procedure, it is modified according to the experts’ comments and the results of the lecture review.

### Quantitative analysis

After constructing a Bayesian belief network, a probabilistic inference is taken to elucidate the dependence relationships between variable nodes in the network. In our study, probabilistic inference was achieved by calculating the joint conditional probability distributions among variables based on their cause and consequence relationships. For each BBN node, a conditional probability formula or table, which represents the probabilities of each value of the node given the conditions of its parents, is supplied. In addition, the distributions of the parent nodes can be calculated given the values of their children. After the conditional probability table is obtained, the joint probability distributions can be decomposed into local probability distributions.

The joint probability distribution formula in BBN can be expressed as


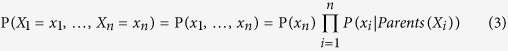


where P(*x*_1_, …, *x*_*n*_) refers to the probability of a specificcombination of values *x*_1_, …, *x*_*n*_ from the set of variables (*X*_1_, …, *X*_*n*_), where Parent (*X*_*i*_) refers to the set of 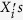 immediateparent nodes. Thus, *P*(*x*_*i*_|*Parents*(*X*_*i*_)) reflects the conditionalprobability, which is related to the node *X*_*i*_ based on its parent nodes.

According to the conditional probability table, the level of outbreak risk with the highest probability was considered the most probable potential risk. In contrast to other outbreak risk analysis methods, BBN provides an index of reliability (i.e., the joint probability distributions of the most probable potential risk).

### Rough Set Method

The rough set method, proposed by Pawlak in 1982^23^, offers mathematical tools to discover hidden patterns in data; therefore, its application in forewarning models needs to be investigated. Objects, which may be indiscernible due to the limited available information, can be expressed by the lower and upper approximations defined based on two extreme cases (full inclusion or nonempty overlay), regarding the relationships between an equivalence class and a target set. The main goal of the rough set-based analysis is to synthesize an approximation of concepts from the acquired data. Because of the novel and unique theory posed by rough set, it has become an important form of intelligence information processing technology that has been widely used in the fields of learning and knowledge discovery, data mining, and decision support and analysis. Rough set theory has also been used successfully in diagnosis and outcome prediction, which is discussed in a large number of articles. Su *et al*.^24^ (2006) used rough set theory to select the relevant features from data to predict diabetes. Wang *et al*.^25^ (2006) applied rough set theory to predict the malignancy degree of brain gliomas and achieved satisfactory results. Bai *et al*.^40^ (2010) utilized rough set to explore spatial decision rules in neural-tube birth defects and predict where high rates of the disease would occur.

Rough set theory is data-driven, and the advantage of this approach is that it does not require the user to make any a priori assumptions about the data. When using this method for epidemiological warnings, the whole analysis process can be divided into four steps: (1) data discretization, which, in this case, in order to ensure the comparability of different models, is the same as the data preparation which has been used in the Bayesian Network model; (2) find minimal reducts of conditional attributes, remove redundant attributes, and reduce system complexity; (3) generate rules according to the reducts, which can provide a rule base for inference and prediction; and (4) apply the rules to predict the epidemic outbreak risk level.

### Linear Regression Model

As a comparison, a traditional linear regression model was used to estimate the HFMD outbreak risks. Regression analysis is a strong scientific method, which is an important branch of modern applied statistics. It has been widely used in many scientific fields. It can not only extract the information hidden in massive raw data groups and grasp the main characteristics of the population data to obtain the mathematical expression of the relationship between variables, but it can also use one or more variables to predict or forecast another dependent variable value. Regression forecasting method is the use of regression analysis method, through the statistical processing and analysis of a large number of observation data, to find out the relationship between the number of predictive objects and influencing factors. The theory of multiple linear regression requires data that generally follows a multivariate normal distribution with the same covariance, but it is usually difficult to meet such conditions with epidemiological data, where a lot of influence factors have 0/1 distribution. At this time, we can use a logistic regression model to predict the disease. For example, the most commonly used predictive method in the study of cancer epidemiology is the logistic regression method.

### Model Evaluation

Model performance was evaluated with receiver operating characteristic (ROC) analysis, which has been widely useful for evaluating the performance of binary classifier. A typical ROC curve plots true positive rate (sensitivity) against false positive rate (1-specificity) for the entire range of possible thresholds, therefore providing a unified representation for assessing the overall model performance. The area under the curve (AUC) was used as a single performance measure to decide whether the model prediction was better than random (0.5). A perfect model would yield an AUC value of 1. Training AUC values were fairly high across models and higher than the testing AUC values, as anticipated (Phillips, 2010). The testing AUC values, which demonstrate the actual model predictive powers, suggest whether the model predictions are better than random.

### Data preparation

The risk of disease outbreak is the probability or chance that a specified disease outbreak will occur. Existing outbreak definitions are composed of two components: (i) an endemic channel that aims to replicate historical trends of expected cases; and (ii) a set of criteria that determines what level of variation above this endemic frequency should be classified as an outbreak^26^. The methods used to define outbreak risk are closely linked to the targets of various disease surveillance methods and to the transmission characteristics of the specified diseases. One common method for defining further disease outbreak risk is to calculate the probability and extent that the current disease occurrence deviates from the historical baseline (i.e., the average value of historical data).

In the existing study, factors that affect the risks of disease outbreak in different areas have been classified as demographic, socioeconomic, and meteorological^27^,^28^. There are many statistical methods used to assess the relationship between risk factors and risks of disease outbreaks, such as principal component analysis and geo-detector^29^. In this study, we performed correlation analysis using SPSS software from IBM. The full dataset (both training and test set) was used to decide which risk factors were relevant.

Because BBNs are optimized for discrete variables, all continuous variables should be discretized before data is put into the network after determining risk factors. At present, there are three primary methods^30^ for discretizing continuous variables: equal intervals, equal frequencies, and entropy. The equal interval method is the simplest unsupervised discretization algorithm; it divides the continuous variable space into a series of average intervals based on the range of numerical attributes^31^. Similarly, the equal frequency method is based on equal numbers of data samples, and it divides the continuous variable space into a plurality of intervals based on the data frequency. The entropy method is supervised from beginning to end. It chooses the minimum entropy value in the numerical attribute as the split point and then classifies the interval results recursively, eventually resulting in layered discretization results. In this study, we used the entropy method to discretize continuous variables.

## Results

To test the feasibility and effectiveness of BBN in assessing infectious disease outbreak risk, we respectively applied BBN to assess the hand, foot, and mouth disease (HFMD) and measles outbreak risks based on disease cases and symptoms at different spatial scales.

### Case one: risk assessment of hand, foot and mouth disease (HFMD) outbreaks

In this case, a HFMD outbreak was defined as an abnormal increase in the severity and mortality of HFMD within the next month. BBN was used to assess HFMD outbreak risk in Hunan Province, China based on virus detection rate and demographic, socioeconomic and meteorological factors. It should be noted that the study period was during the peak months (April–July, Sep–Nov) of HFMD in Hunan^32^, and the study unit was the county.

### Data sources

The disease data incorporated in the in BBN were the rate, severity, and mortality of HFMD and the rate of virus EV71 detection in mild cases. People older than five years were ignored in the study because HFMD mainly infects children under the age of 5^32^. Considering that pre-kindergarten children and kindergarten students have different routes of exposure to virus EV71, we separately calculated the rates of virus EV71 detection in mild cases for children aged 0–3 and 3–5 years old. All HFMD cases and virus detection laboratory data were collected from the local CDC.

The environmental factors in this study were socioeconomic and meteorological factors. The socioeconomic factors included the density of the susceptible population (i.e., children under the age of 5) and urbanization levels. These data were extracted from the *Hunan Statistical Yearbook*^33^,^34^,^35^,^36^,^37^. The meteorological data were obtained from the China Meteorological Data Sharing Service System and included the average air pressure, maximum wind speed, average wind speed, average relative humidity, minimum relative humidity, and average temperature, temperature difference, rainfall, and sunshine.

In this study, the moving percentile method^38^, which has been used successfully in the China Infectious Disease Automated-alert and Response System (CIDARS), was applied to determine the risk levels of HFMD outbreaks. This method assesses the risk of HFMD outbreaks by comparing the reported severity and mortality rates of HFMD during the current observational period to that of the corresponding historical period at the county level. To account for the stability of the data, the current month was used as the current observation period, and the four months prior were used as the historical period. The severity and mortality rates of HFMD in the current observational period were the proportion of severe cases (i.e. cases sent to ICU) and of deaths, respectively, among all reported HFMD cases during the most recent month. We set the percentile of historical data, *P*_50_, *P*_80_, as the indicator of potential outbreak risks. When the severity and mortality rates of HFMD in the current observation period were less than P_50_, the risk level was set as level 0 (low risk). When the rate fell within [P_50_, P_80_], the risk level was level 1(moderate risk). If the rate was more than P_80_, outbreak risk was high, and the risk level was set as level 2. The current observation period and historical data block were dynamically shifted forward by month.

When all data were collected, discretization was performed prior to constructing the network. Table 1 shows each variable classification attribute in the BBN.

### The Application of BBN Model and Its Result

In this study, we used 70% of the data (98 samples) as the training data to build the network, and the remaining 42 samples were used as test data.

**Figure 2:**
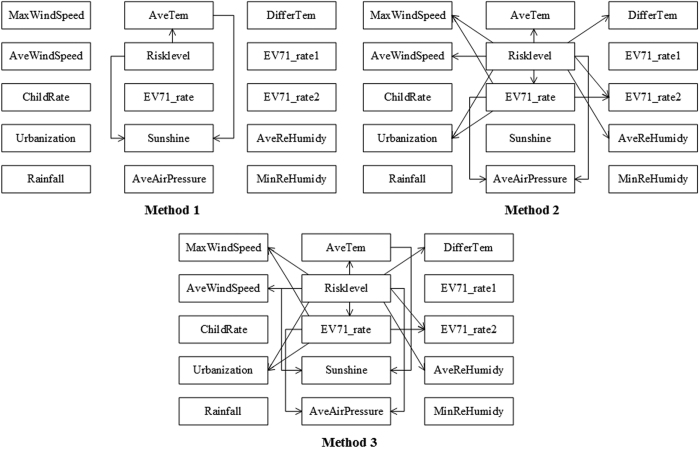
The Bayesian Belief Network used to assess HFMD outbreak risk.

In this case, there were three different ways to construct the Bayesian belief network: (1) only the network structure learning algorithm proposed by Cheng Jie^21^ was used to determine the structure; (2) the network structure was first based on expert knowledge, and then the network structure learning algorithm was applied to modify the network; (3) the expert knowledge method followed the network structure learning algorithm in the course of network building. Figure 2 illustrates the Bayesian belief network construction via these three methods. In all networks, the variables are represented as nodes, and the arcs between the nodes indicate the relationships between variables. In addition, the arrows on the arcs extend from nodes to child nodes^39^.

**Figure 3:**
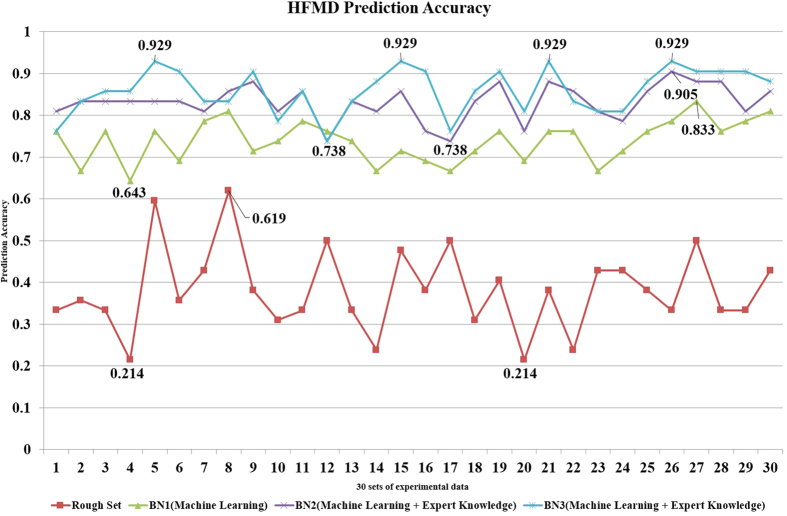
The accuracy of the BBN and rough set method used to assess HFMD outbreak risk.

To evaluate the performances of different Bayesian belief networks, the percentage of samples correctly predicted from all test samples was taken as the index of the risk assessment accuracy. Figure 3 shows the risk assessment accuracy of these three networks. As seen in Fig. 3, all three networks performed well in assessing the HFMD outbreak risk. In addition, the risk assessment accuracy of the third method was highest. The highest accuracy value reached via the third method over the course of 30 experiments was 92.90 ± 4.61% at the 95% confidence level. Therefore, the networks constructed by the third method were finally applied to assess the HFMD outbreak risks in the study area. This network structure shows that the risk level was dependent on the rate of virus EV71 detection(r = 0.125, p = 0.009), urbanization levels(r = 0.275, p = 0.011), rate of virus EV71 detection in mild cases in children aged 3–5 years old(r = −0.119, p = 0.012), average air pressure(r = 0.098, p = 0.038), average relative humidity(r = 0.119, p = 0.011), average wind speed(r = −0.120, p = 0.000), maximum wind speed(r = −0.029, p = 0.037), average temperature(r = −0.268, p = 0.000), and temperature difference (r = 0.192, p = 0.000).

As seen in the conditional probability table (Table 2), the effect of HFMD outbreak risk varied among the selected factors. Following the increase in the rate of virus EV71 detection, especially in mild cases in children aged 3–5 years old, the risk of HFMD outbreak appeared to increase. Some environmental factors also impacted the HFMD outbreak risk. When the average relative humidity was over 77.765%, the probability of risk level 0 suddenly decreased to 0.043, but the probability of risk level 2 remained at 0.25. The average temperature was found to have a negative effect on the HFMD outbreak risk; an HFMD outbreak was more likely to occur in lower temperatures.

### The Application of Rough Set Method and Its Result

To compare with the risk assessment accuracy of the rough set method with BBN, we applied the rough set method in the HFMD and measles outbreak risks prediction experiments. Both the training data and the test sample data were input to each method. In the rough set method, we obtained 179 reducts from the decision system. The smallest set contains four attributes: the rate of virus EV71 detection in mild cases for children aged 0–5 years old, urbanization level, average temperature, and average wind speed. This means that at least four spatial attributes should be used to synthesize the decision rules. According to the 179 reducts, we next generated 17,165 rules. These rules, which discovered the relationship between the attributes and decision, are the decision rules. According to the decision rules, the risk levels of the HFMD outbreak were estimated. Figure 3 shows the accuracy of the BBN and the rough set method in 30 different experiments of the HFMD outbreak risk analysis. The prediction accuracy of the BBN, which fluctuated between 0.929 and 0.738, is markedly higher than that of the rough set method, which fluctuated between 0.619 and 0.214 in the experiments.

### The Application of Linear Regression Model and Its Result

As a comparison, we also used a tradition linear regression model to estimate the HFMD outbreak risk. In this case, as the dependent variable is a discrete classification, we used the logistic regression model, which is the most commonly used method in epidemiological prediction research, to predict the HFMD outbreak risk level (0, 1, or 2). As was done before when constructing Bayesian Belief Networks and using Rough Set methods, the training data were input to build the logistic regression model, and the test data were criterion. The 30 experiments were carried out, and 30 fitting results were obtained. When we modelled each data set, we chose the optimal logistic model through the value of R^2^. In these statistic models, the general form was as follows:


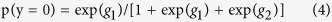



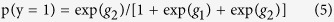



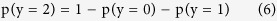


Where


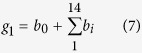



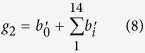


Among them, p indicates the probability of the HFMD outbreak risk level; *b*_0_ and 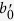 are constant terms; *b*_*i*_ and 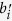 are the coefficients, which correspond to different levels of various discrete risk factors x_i._

### Case two: Measles outbreak risk analysis

In this case, a measles outbreak was defined as an outbreak on the verge of happening. The goal of this case was to assess the measles outbreak risk in regions of China between December 1^st^, 2004 and June 30^th^, 2007, by BBN. The study area falls between 25°N–30°N and 105°E–115°E, China. The study unit in this case was the town.

### Data sources

Data from 438 disease cases were used in this study and were provided by the China CDC. In addition to outbreak data, the disease data included records of measles cases at the specified regions in the study area, although no outbreak had been reported simultaneously.

According to the transmission characteristics of measles, the risk factors used here included residence environment factor and meteorological factors. The residence environment factor was whether there was a school or a factory in the town. The meteorological data came from the China Meteorological Data Sharing Service System and included diurnal barometric range, minimum daily temperature, daily amount of sunshine, minimum daily humidity, and maximum daily wind speed.

All meteorological data were calculated by the Inverse Distance Weighting (IDW) method based on values from 756 national meteorological stations. All continuous variables were discretized before being entered into the Bayesian network.

### The Application of BBN Model and Its Result

**Figure 4:**
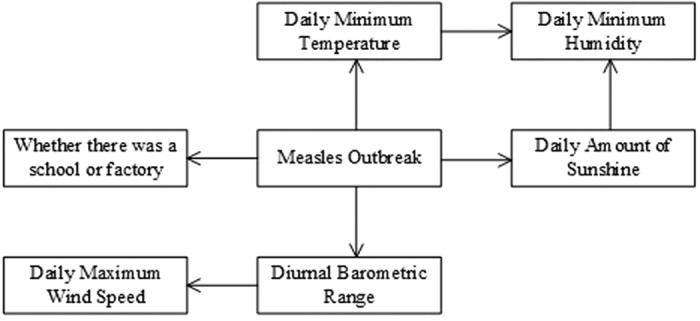
The Bayesian Belief Network used to assess measles outbreak risk.

In this case, the network structure learning algorithm was applied to construct the network, and then the network structure was modified based on expert knowledge. As seen in Fig. 4, the risk level of a measles outbreak was a father node of the residence environment factor, daily minimum temperature, diurnal barometric range, and daily amount of sunshine. The diurnal barometric range depended on the daily maximum wind speed. In addition, both the daily minimum temperature and daily amount of sunshine were child nodes of daily minimum humidity.

Table 3 shows the conditional probability distribution for the risk level of a measles outbreak based on seven factors. As seen in Table 3, measles outbreaks were more likely in crowded places, such as schools and factories, with a probability of 0.9469. When the minimum daily temperature was −2.675–8.65 °C, the outbreak probability was also high, occasionally reaching 0.9125. By contrast, when the minimum daily temperature was >19.975 °C, the outbreak probability was lowest, at 0.0255. The table also shows that the daily amount of sunshine had no influence on measles outbreaks; the probability fluctuated between 0.0745 and 0.1946. Researchers could use this impact factor data to make inferences about measles outbreak risk based on the table. For example, when the daily minimum temperature was 0.5 °C, the probability of a measles outbreak could be as high as 0.9125.

### The Application of Rough Set Method and Its Result

**Figure 5:**
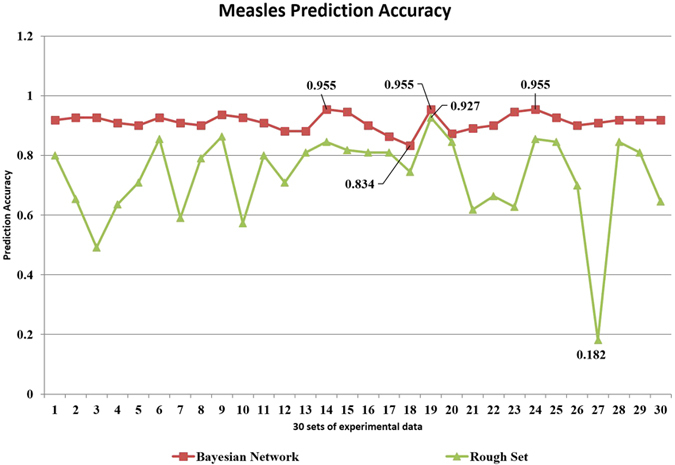
The accuracy of the BBN and the rough set method used to assess measles outbreak risk.

As a comparison, we also applied the rough set method to assess measles outbreak risk in the same area during the same period. In the BBN, linear regression model, and rough set method, 75% of the sample data (328) were randomly selected as the training data, and the remaining 110 samples served as the test data. To verify the accuracy and reliability of the method, we repeated the random sampling process 30 times, producing 30 sets of experimental data. The results (seen in Fig. 5) showed that the assessment accuracy of the measles outbreak risk for the BBN distributed at (1, 0.8). The highest accuracy value was 95.50 ± 3.50% at the 95% confidence level. In brief, the distance between the BBN and rough set method with regard to the accuracy of their assessments of measles outbreak risk was short. However, the fluctuations in the value of assessment accuracy of the rough set method reached large amplitudes. In 30 experiments, the highest accuracy of the rough set method reached 0.927, but the lowest accuracy was only 0.182. In the experiment with the highest accuracy, only one result containing all six attributes (daily minimum temperature, daily minimum humidity, daily amount of sunshine, diurnal barometric range, daily maximum wind speed, and whether there was a school or a factory in the town) was obtained. Then, 56 rules describing the relationship between the attributes and the decision were generated.

### The Application of Linear Regression Model and Its Result

Similarly, we used logistic regression model to predict the measles outbreak risk level. The theory and procedure of how to use it were the same as the HFMD case before. The general form of models that be simulated from 30 sets experimental data was as follows:


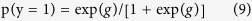


Where


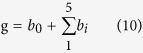


Among them, p represents the probability of measles outbreak risk; *b*_0_ is a constant term; *b*_*i*_ is the coefficient which corresponds to different levels of discrete various discrete risk factors x_i_.

### The Evaluation of the BNN model, Rough Set Method and Linear regression model

**Figure 6:**
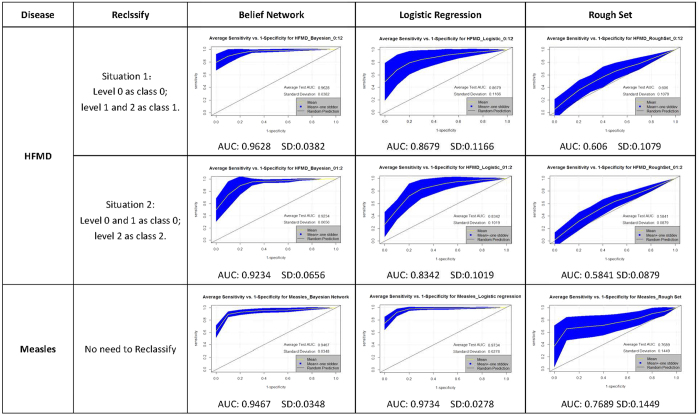
ROC curve for Bayesian Belief Network model, Logistic Regression model and Rough Set method prediction averaged on each 30 replicate runs. (The 1:1 line indicates the condition if the prediction is completely out of random chance (AUC = 0.5)).

Because the ROC curve applies only to binary classifiers, we had to reclassify the HFMD outbreak risk level (0 or 1 or 2) into two classes. Situation 1: we defined level = 0 as the class that means there is no HFMD outbreak, and we merged level = 1 and level = 2 as the other class to represent the outbreak; Situation 2: we merged level = 0 and level = 1 to represent that the epidemic is not serious, and level = 2 as a representation of a very high HFMD outbreak risk. Meanwhile, for the measles dataset, due to the outbreak risk level (0 or 1) set as two values, reclassification was not needed. The ROC curves for each model’s average prediction of 30 replicate runs with a mean test AUC value and corresponding standard deviation are provided in Fig. 6.

In the lateral view, for the HFMD test dataset, no matter how to reclassify the risk level, the Bayesian network model achieved a very high performance evaluation (the average AUC test was 0.9628 for situation 1 and 0.9234 for situation 2), and better than the logistic regression model (average AUC test was 0.8679 for situation 1 and 0.8342 for situation 2) and rough set method (average AUC test was 0.606 for situation 1 and 0.5841 for situation 2). In the measles test dataset, the Bayesian network model (average test AUC was 0.9467) and logistic regression model (average test AUC is 0.9734) showed high prediction accuracy and were obviously superior to the rough set method (average AUC test was 0.7689). In the longitudinal view, the stability of the Bayesian network is good (standard deviation was 0.0382 for HFMD situation1, 0.0656 for HFMD situation2, and 0.0348 for measles), regardless of the classification of the disease, and the logistic regression model (standard deviation was 0.1166 for HFMD situation1, 0.1019 for HFMD situation2, and 0.0278 for measles) and rough set method (standard deviation was 0.1079 for HFMD situation1, 0.0879 for HFMD situation2, and 0.1449 for measles) do not have this advantage.

## Discussion

In recent years, early warning systems have played an important role in preventing and controlling the spread of infectious diseases. However, the recent requirement for very early detection has shifted in the direction of research. An increasing number of studies focused on improving the speed and accuracy of outbreak risk assessment have been conducted. In this article, a general approach integrating the Bayesian network concept with infectious disease risk assessment was proposed and demonstrated. We first introduced the methodology for assessing the disease outbreak risk based on a BBN, and then detailed how to assess the outbreak risks of measles and HFMD using a BBN based on disease cases and symptoms at different spatial scales. Finally, we compared the accuracy and feasibility of the BBN and rough set methods. Our results showed that the BBN performed better than the rough set method, both in terms of accuracy and stability. Moreover, once the relationship network among disease outbreak risks and factors was determined, the BBN accurately predicted the further risk of infectious disease outbreaks without several selected factors.

There are many advantages to using a BBN to assess disease outbreak risk^24^. Firstly, a BBN describes the causal probability relationship between disease outbreak risk and risk factor variables, including variability, uncertainty, and subjectivity^25^. Therefore, the accuracy of risk assessment is not influenced by the missing variables. Secondly, because a BBN is a graph, it is easy to update and to understand the relationships among variables. In a BBN, the probabilistic causal dependencies among variables are expressed by arcs, and the strength of the dependencies are displayed in probability distribution tables. Lastly, according to the joint probability distributions of various disease outbreak risk levels, a BBN not only determines the most probable risk level, but also gives the probability that this risk level will appear and provides a detailed risk assessment to the public health department for disease surveillance. Although this study only applied a BBN to assess the outbreak risks of HFMD and measles, it can also be used to simultaneously assess the outbreak risk for other similar diseases.

Nevertheless, use of a BBN to assess infectious disease outbreak risks presents some limitations. Because a BBN is optimized to handle categorical variables, continuous data often must be discretized before modelling, which may result in information loss. Moreover, the accuracy of the method is closely related to the network structure. Although there are many network construction algorithms, it is difficult to update a proper Bayesian belief network in automatic and timely manner according to changes in the disease transmission process. This will affect the accuracy and sensitivity of disease outbreak risk assessment. In addition, the risk factors selected in this study were limited, and more related risk factors, such as symptom surveillance data and reported data from specified organizations, should be involved in future studies.

## Additional Information

**How to cite this article**: Liao, Y. *et al*. A new method for assessing the risk of infectious disease outbreak. *Sci. Rep.* **7**, 40084; doi: 10.1038/srep40084 (2017).

**Publisher's note:** Springer Nature remains neutral with regard to jurisdictional claims in published maps and institutional affiliations.

## Figures and Tables

**Table 1:** Input variables in the BBN for estimating HFMD outbreak risk.

Variables name	Corresponding categorical values	Initial values	Variables name	Corresponding categorical values	Initial values
The Risk Level of HFMD outbreak	Low	<P50	Average Relative Humidity (%)	Low	<70.850
	Intermediate	P50-P80		Lower Intermediate	70.850–73.994
	High	>P80		Intermediate	73.994–75.727
The rate of virus EV71 detection in mild case for children aged 0–5 years old (1/100)	Low	<0.057		Upper Intermediate	75.727–77.765
	Lower Intermediate	0.057–0.151		High	>77.765
	Intermediate	0.151–0.267	Average Temperature (°C)	Low	<18.283
	Upper Intermediate	0.267–0.407		Lower Intermediate	18.283–21.508
	High	>0.407		Intermediate	21.508–23.229
The rate of virus EV71 detection in mild case for children aged 0–3 years old (1/100)	Low	<0.050		Upper Intermediate	23.229–27.321
	Lower Intermediate	0.050–0.155		High	>27.321
	Intermediate	0.155–0.263	Average Wind Speed (m/s)	Low	<1.658
	Upper Intermediate	0.263–0.415		Lower Intermediate	1.658–1.797
	High	>0.415		Intermediate	1.797–1.958
The rate of virus EV71 detection in mild case for children aged 3–5 years old (1/100)	Low	<0.05		Upper Intermediate	1.958–2.194
	Lower Intermediate	0.05–0.354		High	>2.194
	Upper Intermediate	0.354–0.9	Minimum Relative Humidity (%)	Low	<49.535
	High	>0.9		Lower Intermediate	49.535–53.342
The density of the susceptible population (ie., children under the age of 5) (pop/km^2^)	Low	<20.299		Intermediate	53.342–55.675
	Lower Intermediate	20.299–21.081		Upper Intermediate	55.675–58.935
	Intermediate	21.081–29.716		High	>58.935
	Upper Intermediate	29.716–33.449	Rainfall (mm)	Low	<3.017
	High	>33.449		Lower Intermediate	3.017–3.752
Average air pressure (hPa)	Low	<982.689		Intermediate	3.752–5.751
	Lower Intermediate	982.689–987.763		Upper Intermediate	5.751–7.574
	Intermediate	987.763–994.911		High	>7.574
	Upper Intermediate	994.911–1003.306	Sunshine Hours (h)	Low	<21.916
	High	>1003.306		Lower Intermediate	21.916–30.908
Urbanization Level (%)	Low	<42.815		Intermediate	30.908–41.319
	Lower Intermediate	42.815–48.555		Upper Intermediate	41.319–52.896
	Intermediate	48.555–56.825		High	>52.896
	Upper Intermediate	56.825–69.78	Temperature Difference (°C)	Low	<7.213
	High	>69.78		Lower Intermediate	7.213–7.547
Maximum Wind Speed (m/s)	Low	<3.934		Intermediate	7.547–8.029
	Lower Intermediate	3.934–4.222		Upper Intermediate	8.029–8.544
	Intermediate	4.222–4.499		High	>8.544
	Upper Intermediate	4.499–4.943			
	High	>4.943			

**Table 2:** Conditional probability distribution table of the BBN for estimating HFMD outbreak risk.

Variables name	Corresponding categorical values	Probability of differentrisk levels			Variables	Corresponding categorical values	Probability of different risk levels		
		0	1	2			0	1	2
The rate of virus EV71 detection in mild case for children aged 0–5 years old	Low	0.348	0.214	0.1	Average Relative Humidity	Low	0.13	0.214	0.2
	Lower Intermediate	0.087	0.286	0.2		Lower Intermediate	0.261	0.143	0.2
	Intermediate	0.174	0.071	0.15		Intermediate	0.261	0.071	0.1
	Upper Intermediate	0.043	0.214	0.3		Upper Intermediate	0.304	0.286	0.25
	High	0.348	0.214	0.25		High	0.043	0.286	0.25
The rate of virus EV71 detection in mild case for children aged 3–5 years old	Low	0.426	0.335	0.278	Average Temperature	Low	0.043	0.214	0.35
	Lower Intermediate	0.288	0.244	0.253		Lower Intermediate	0.043	0.071	0.3
	Upper Intermediate	0.158	0.208	0.318		Intermediate	0.261	0.357	0.15
	High	0.129	0.213	0.151		Upper Intermediate	0.174	0.286	0.1
Average air pressure	Low	0.226	0.274	0.146		High	0.478	0.071	0.1
	Lower Intermediate	0.183	0.172	0.176	Average Wind Speed	Low	0.043	0.214	0.15
	Intermediate	0.161	0.172	0.272		Lower Intermediate	0.261	0.143	0.2
	Upper Intermediate	0.241	0.172	0.26		Intermediate	0.087	0.286	0.25
	High	0.19	0.208	0.146		Upper Intermediate	0.261	0.286	0.15
Urbanization Level	Low	0.226	0.274	0.174		High	0.348	0.071	0.25
	Lower Intermediate	0.212	0.208	0.198	Temperature Difference	Low	0.217	0.143	0.2
	Intermediate	0.248	0.203	0.291		Lower Intermediate	0.217	0.143	0.2
	Upper Intermediate	0.154	0.142	0.217		Intermediate	0.304	0.214	0.25
	High	0.161	0.172	0.121		Upper Intermediate	0.174	0.357	0.2
Maximum Wind Speed	Low	0.226	0.274	0.174		High	0.087	0.143	0.15
	Lower Intermediate	0.212	0.208	0.198					
	Intermediate	0.248	0.203	0.291					
	Upper Intermediate	0.154	0.142	0.217					
	High	0.161	0.172	0.121					

**Table 3:** The conditional probability distribution table of the BBN for estimating measles outbreak risk.

Variable name and value	The probability of an outbreak		Variable name and value	The probability of an outbreak		Variable name and value	The probability ofan outbreak	
Whether there is a school or a factory	No	Yes	Daily maximum wind speed	No	Yes	Daily sunshine time	No	Yes
No	0.9362	0.0639	Low	0.8811	0.1189	Low	0.8057	0.1946
Yes	0.0536	0.9469	Intermediate	0.4724	0.5276	Lower Intermediate	0.8986	0.1019
			High	0.5732	0.4268	Upper Intermediate	0.8722	0.1279
						High	0.9255	0.0745
**Barometric diurnal range**	**No**	**Yes**	**Daily minimum temperature**	**No**	**Yes**	**Daily minimum humidity**	**No**	**Yes**
Low	0.9152	0.0848	Low	0.4724	0.5276	Low	0.5417	0.4583
Lower Intermediate	0.6055	0.3945	Lower Intermediate	0.0875	0.9125	Lower Intermediate	0.9095	0.0905
Upper Intermediate	0.3092	0.6908	Upper Intermediate	0.4315	0.5685	Upper Intermediate	0.9030	0.0970
High	0.4724	0.5276	High	0.9745	0.0255	High	0.8501	0.1499
